# Characterization of the complete chloroplast genome of *Euphorbia pekinensis* Rupr. (Euphorbiaceae)

**DOI:** 10.1080/23802359.2022.2111978

**Published:** 2022-08-26

**Authors:** Yu-Liang Wang, Xing Jian, Song Wang

**Affiliations:** a College of Life and Healthy Science, Anhui Science and Technology University, Bengbu, PR China; b College of Architecture, Anhui Science and Technology University, Bengbu, PR China

**Keywords:** *Euphorbia pekinensis*, Euphorbiaceae, chloroplast genome, phylogeny

## Abstract

*Euphorbia pekinensis* Rupr. 1859 is a medicinal herb endemic to China and distributed throughout the country, particularly across the northern part of the mainland. However, the systematic classification of Euphorbiaceae remains controversial. Therefore, studying the chloroplast genome of *E. pekinensis* is crucial for the resolution of this taxonomic dispute, clarification of the systematic status of *Euphorbia*, and establishment of an accurate classification system for Euphorbiaceae. In this study, we sequenced the complete chloroplast genome of *E. pekinensis* using Illumina sequencing technology and annotated it using GeSeq. The complete chloroplast genome was 162,002-bp-long with a guanine–cytosine (GC) content of 35.7%. It included one large single-copy (LSC), one small single-copy (SSC), and two inverted repeat sequence regions (IRa and IRb), which were 90,225 bp, 18,067 bp, and 26,855 bp in length, respectively, and are indicative of a typical tetrad structure. The genome encoded 129 functional genes, comprising 85 protein-coding genes, 36 tRNA genes, and eight rRNA genes. According to the maximum-likelihood phylogenetic tree that was constructed using 16 complete chloroplast genomes, *E. pekinensis* was found to be closely related to *E. ebracteolata*. Therefore, the complete chloroplast genome of *E. pekinensis* provides a better understanding of *Euphorbia* genetics.

*Euphorbia pekinensis* belongs to *Euphorbia*, Euphorbiaceae; this genus includes over 2000 species with the shared characteristics of milky sap and cyathium. *Euphorbia* is among the largest angiosperm genera and is distributed worldwide, particularly in Africa and Central and South America (Zhang et al. 2020). Many *Euphorbia* species, including *E. pekinensis*, are used as medicinal plants. The dried roots of the plant contain compounds such as diterpenoids, triterpenoids, flavonoids, and tannins. *Euphorbia pekinensis* has been widely used in clinical applications to treat edema, abdominal distension, hydrothorax, phlegm-fluid retention, and other diseases. However, the classification of Euphorbiaceae remains controversial. The phylogenetic relationships among species of *Euphorbia* have yet to be determined. Euphorbiaceae genera have been divided according to their morphological traits into four subfamilies: Phyllanthoideae, Acalyphoideae, Crotonoideae, and Euphorbioideae. In the Angiosperm Phylogeny Group IV system (The Angiosperm Phylogeny Group 2016), Euphorbiaceae *sensu lato* was divided into Phyllanthaceae, Putranjivaceae, Euphorbiaceae *sensu stricto*, and Peraceae. Among the Euphorbiaceae, *Euphorbia* has the highest species diversity and widest distribution area (Shen 1998). Some botanists believe that this genus should be divided into several subgenera (Steinmann and Porter 2002). The phylogenetic relationships among species of *Euphorbia* have yet to be determined. Therefore, there is a need to further investigate this genus. The study of the complete chloroplast genome of *E. pekinensis* would aid in determining its evolutionary position.

Fresh leaves of *E. pekinensis* were collected from Fengyang County, Anhui Province, China (117.5598°E, 32.8816°N) and dried using silica gel. A specimen was deposited at the Herbarium of the Anhui Science and Technology University under the voucher number AHSTU003289 (www.ahstu.edu.cn, Yu-Liang Wang, roystonea@163.com). Its genomic DNA was extracted using the cetyltrimethylammonium bromide method (Doyle and Doyle 1987). The extracted DNA was sent to Nanjing Genepioneer Biotechnology Co., Ltd. (Nanjing, China) to construct a DNA library, which was sequenced using the Illumina HiSeq 4000 sequencing platform (Illumina, San Diego, CA). Reads of the complete chloroplast genome were assembled using *de novo* assembly in GetOrganelle (Jin et al. 2020) with k-mer lengths of 21–105 bp, followed by reference-guided assembly performed using Bandage 0.8.1 (Wick et al. 2015). *Euphorbia maculata* (GenBank accession number: NC_052745.1) genome was used as a reference for the annotation using GeSeq (Tillich et al. 2017); this was coupled with manual correction for boundaries. A circular chloroplast genome map was constructed using the OGDRAW program (Greiner et al. 2019). To identify the phylogenetic position of *E. pekinensis*, a maximum-likelihood phylogenetic tree was constructed using the complete chloroplast genomes of 16 species using IQ-Tree 2 (Bui et al. 2020).

The complete chloroplast genome of *E. pekinensis* was 162,002-bp-long (GenBank accession number: MZ707776) with a guanine–cytosine (GC) content of 35.7%. The genome contained one large single-copy, one small single-copy, and two inverted repeat (IR) sequence regions which were 90,225 bp, 18,067 bp, and 26,855 bp in length, respectively. There were 129 functional genes in total, comprising 85 protein-coding genes, 36 tRNA genes, and eight rRNA genes. Eight protein-coding genes, seven tRNA genes, and four rRNA genes were duplicated within the IR regions. Moreover, 11 genes in the chloroplast genome contained introns, of which nine genes contained one intron and two genes contained two introns.

A phylogenetic tree was constructed using the complete chloroplast genomes of *E. pekinensis* and 14 other species from the family Euphorbiaceae, which were downloaded from the National Center for Biotechnology Information repository, whereas the chloroplast genome of *Buxus microphylla* (Buxaceae) functioned as an outgroup (Figure 1). The results showed that all six species of *Euphorbia* formed a monophyletic clade. Among them, *E. pekinensis* and *Euphorbia ebracteolata* formed a clade with a high bootstrap support (100%), which shared a sister relationship with *Euphorbia esula*. Therefore, this complete chloroplast genome of *E. pekinensis* will enable the scientific community to better comprehend *Euphorbia* genetic information.

**Figure 1.**
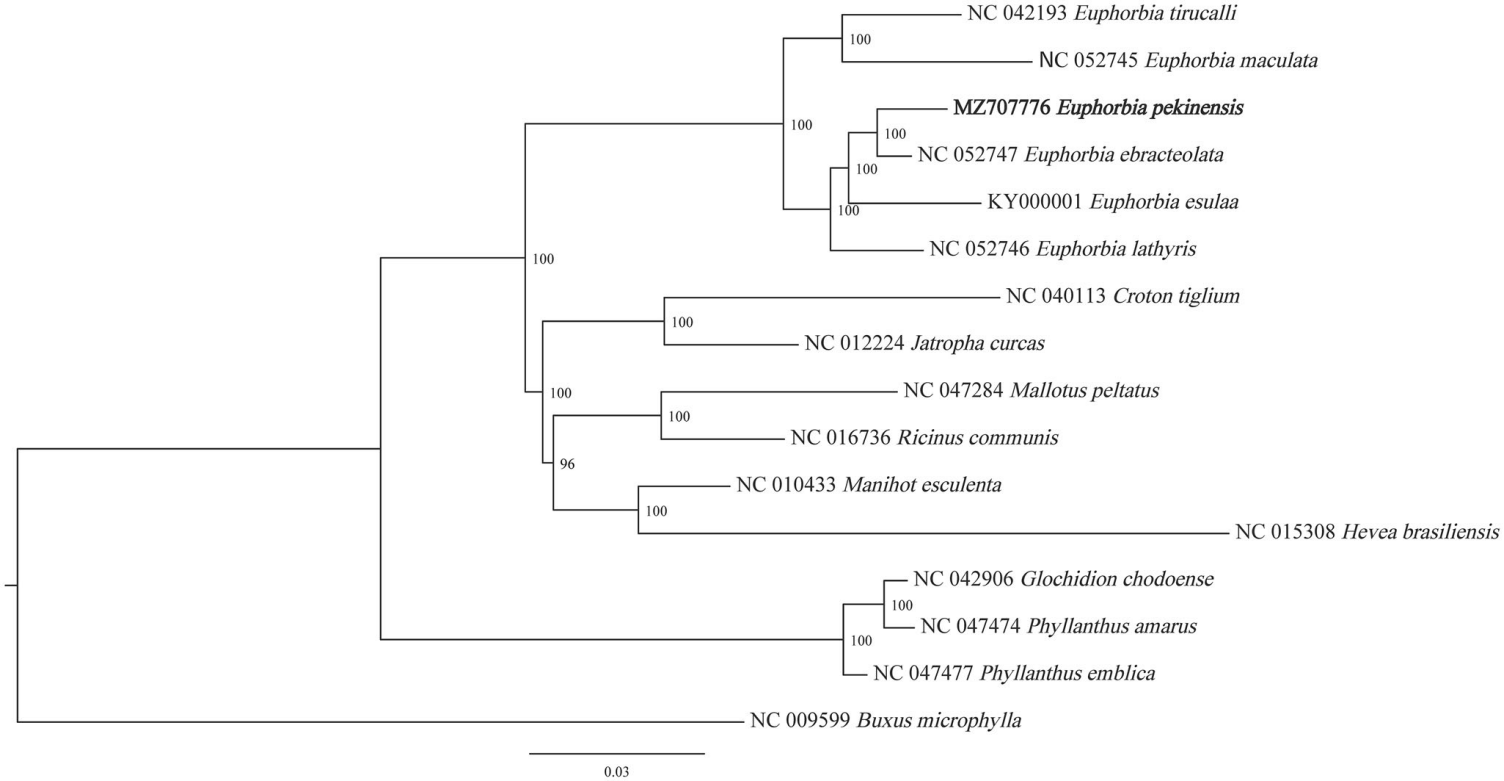
Maximum-likelihood phylogenetic tree based on complete chloroplast genomes of the 16 investigated species (bootstrap repeat is 1000).

## Data Availability

The genome sequence data that support the findings of this study are openly available in GenBank of NCBI at https://www.ncbi.nlm.nih.gov/ under the accession no. MZ707776. The associated BioProject, SRA, and Bio-Sample numbers are PRJNA757126, SRR15570202, and SAMN20934138, respectively.
